# Corrigendum

**DOI:** 10.1093/jxb/erv443

**Published:** 2015-10-08

**The role of cis-zeatin-type cytokinins in plant growth regulation and mediating responses to environmental interactions**

Martin Schäfer^1^, Christoph Brütting^1^, Ivan David Meza-Canales^1^, Dominik K. Großkinsky^2^, Radomira Vankova^3^, Ian T. Baldwin^1^ and Stefan Meldau^4^,*

^1^ Department of Molecular Ecology, Max-Planck-Institute for Chemical Ecology, Hans-Knöll-Str.8, 07745 Jena, Germany

^2^ Department of Plant and Environmental Sciences, Copenhagen Plant Science Centre, University of Copenhagen, Højbakkegård Allé 13, 2630 Taastrup, Denmark

^3^ Laboratory of Hormonal Regulations in Plants, Institute of Experimental Botany AS CR, v.v.i., Rozvojová 263, 165 02 Prague 6, Czech Republic

^4^ KWS SAAT AG, Molecular Physiology (RD-ME-MP), Grimsehlstrasse 31, 37555 Einbeck, Germany

* To whom correspondence should be addressed. E-mail: stefan.meldau@kws.com

Journal of Experimental Botany (2015) **66** (16): 4873–4884 doi:10.1093/jxb/erv214

In the above article, the following text under heading ‘Roles of cZ in plant growth’:

‘Decreasing the amount of cZ by overexpressing cZOGTs in rice delayed leaf-senescence and led to short root phenotypes and bigger number of crown roots (Kudo *et al*., 2012).’

should have read:

‘Decreasing the amount of cZ by overexpressing cZOGTs in rice delayed leaf-senescence and led to short shoot phenotype and decreased number of crown roots (Kudo *et al*., 2012).’

